# Efficacy and safety of artemether-lumefantrine and dihydroartemisinin-piperaquine in the treatment of uncomplicated *Plasmodium falciparum* malaria in Kenyan children aged less than five years: results of an open-label, randomized, single-centre study

**DOI:** 10.1186/1475-2875-13-33

**Published:** 2014-01-28

**Authors:** Bernhards R Ogutu, Kevin O Onyango, Nelly Koskei, Edgar K Omondi, John M Ongecha, Godfrey A Otieno, Charles Obonyo, Lucas Otieno, Fredrick Eyase, Jacob D Johnson, Raymond Omollo, Douglas J Perkins, Willis Akhwale, Elizabeth Juma

**Affiliations:** 1 Centre for Clinical Research, Kenya Medical Research Institute, Kisumu, Kenya; 2 Walter Reed Project/Centre for Clinical Research, Kenya Medical Research Institute, Kisumu, Kenya; 3 Centre for Global Health Research, Kenya Medical Research Institute, Kisumu, Kenya; 4 Drugs for Neglected Disease Initiative, Kenya Medical Research Institute, Nairobi, Kenya; 5 Center for Global Health, University of New Mexico, New Mexico, USA; 6 Department of Disease Control and Prevention, Ministry of Public Health and Sanitation, Nairobi, Kenya; 7 Division of Malaria Control, Ministry of Public Health and Sanitation, Nairobi, Kenya

**Keywords:** Artemether-lumefantrine, Dihydroartemisinin-piperaquine, Uncomplicated *Plasmodium falciparum* malaria

## Abstract

**Background:**

This open-label, randomized study evaluated efficacy and safety of artemether-lumefantrine (AL) and dihydroartemisinin-piperaquine (DP) in treatment of uncomplicated falciparum malaria in children below five years of age, to build evidence on use of AL as first-line treatment and DP as second-line treatment in Kenya.

**Methods:**

A total of 454 children aged six to 59 months with uncomplicated falciparum malaria were randomized (1:1) to receive AL dispersible or DP paediatric tablets and followed up for 42 days. Primary efficacy variable was corrected adequate clinical and parasitological response (ACPR) rate on day 28. Secondary variables included corrected (day 14, 28 and 42), uncorrected (day 3, 14, 28 and 42) cure rates, parasitological failure at days 3, 14 and 42. Acceptability and tolerability of both drugs were assessed by caregiver questionnaire.

**Results:**

On day 28, corrected ACPR rates for AL dispersible and DP paediatric were 97.8% (95% CI: 94.9-99.3) and 99.1% (95% CI: 96.8-99.9), respectively, in intention-to-treat population, with no significant treatment differences noted between AL dispersible and DP paediatric arms. Additionally, no significant differences were observed for PCR corrected cure rates on days 14 and ACPR on day 42 for AL dispersible (100%; 96.8%) and DP paediatric (100%; 98.7%). Similarly, for PCR uncorrected cure rates, no significant differences were seen on days 3, 14, 28, and 42 for AL dispersible (99.1%; 98.7%; 81.1%; 67.8%) and DP paediatric (100%; 100%; 87.7%; 70.5%). Parasite clearance was rapid, with approximately 90% clearance achieved in 40 hours in both treatment arms. Incidence of adverse events was related to underlying disease; malaria being reported in both treatment arms. One serious adverse event was noted in AL dispersible (0.42%) arm, not related to study drug. Adherence to treatment regimen was higher for children treated with AL dispersible (93.6%) compared to DP paediatric (85.6%). Acceptability of AL dispersible regimen was assessed as being significantly better than DP paediatric.

**Conclusions:**

AL and DP were both efficacious and well tolerated, and had similar effects at day 42 on risk of recurrent malaria. No signs of *Plasmodium falciparum* tolerance to artemisinins were noted.

**Trial registration:**

PACTR201111000316370.

## Background

Malaria continues to be a major health problem worldwide, with an estimated 216 million cases in 2010, approximately 174 million of which were in the African region [1]. Children less than five years of age are at increased risk of *Plasmodium falciparum* malaria, with a reported 86% mortality rate globally [1]. Artemisinin-based combination therapy (ACT) is recommended as the first-line treatment for uncomplicated *P. falciparum* malaria by the World Health Organization (WHO) [2], over chloroquine and sulpha drugs worldwide. Multidrug resistance has been reported for monotherapy (for example artemisinin [3-5]) and some of the available combination chemotherapy (for example, sulphadoxine/sulphalene-pyrimethamine [6]) used for malaria. Treatment with ACT is known to improve cure rates, results in rapid parasite clearance [2,7] and reduces gametocyte carriage resulting in a decrease in parasite transmission [2,8-10].

Artemether-lumefantrine (AL, Coartem®, Novartis Pharma AG) is a first fixed-dose ACT, which meets the WHO prequalification criteria for efficacy, safety and quality, and is indicated for the treatment of uncomplicated falciparum malaria, or mixed infections including *P. falciparum* in adults, children, and infants (>5 kg body weight). Since the prequalification of Coartem^®^, other AL generics have been prequalified by WHO. In clinical studies, AL demonstrated consistent 28-day polymerase chain reaction (PCR)-corrected cure rates of more than 95% in adult and paediatric populations with a favourable safety and tolerability profile [11]. With rapid parasite clearance and gametocyte reduction also being reported [11], the widespread adoption of AL has led to a significant contribution in the reduction of malaria burden in Sub-Saharan Africa [12]. Since the Kenyan Ministry of Health introduced AL as the first-line treatment for uncomplicated falciparum malaria in 2006, there has been a notable reduction in child mortality rates [13]. Novartis launched the paediatric formulation of AL (AL dispersible) in 2009, developed jointly with Medicines for Malaria Venture (MMV). It is a sweet-tasting and easy-to-administer tablet which is now approved in over 40 countries. AL dispersible has proven in clinical trials to be as well tolerated and as effective as regular AL tablets [14,15]. Moreover, it has been shown that standard African diets (generally consisting of a carbohydrate staple supplemented by pulses, nuts, meat or fish and fat from oil crops or from vegetables or plants) and milk are adequate to ensure AL efficacy [16]. However, AL is associated with a high risk of re-infection soon after therapy in high-transmission areas [17].

Dihydroartemisinin-piperaquine (DP: Duo-cotecxin®, Holley-Cotec Pharmaceuticals) is an ACT that is administered as a single daily dose over three days and has been shown to be well tolerated and highly effective against falciparum malaria [18]. In a previous comparative assessment, DP has been proven to be as efficacious and safe as AL in the treatment of uncomplicated falciparum malaria in Zambian children less than five years of age. DP has been suggested for use as a rescue and/or alternative treatment to AL, or as second-line treatment following treatment failure with the initial treatment [19]. The Kenyan Ministry of Health adopted DP as second-line treatment in 2009.

To further build on the evidence from earlier studies that used AL as a first-line and DP as second-line treatment, this study evaluated the efficacy and safety of AL dispersible *vs* DP paediatric in Kenyan children less than five years of age for the treatment of uncomplicated falciparum malaria.

## Methods

This was an open-label, randomized, single-centre study (Trial registration number: PACTR201111000316370). The protocol was approved by the Kenya Medical Research Institute Ethics Review Committee. The study was conducted in accordance with the Declaration of Helsinki (2002), Good Clinical Practices guidelines set up by the International Conference on Harmonization [20], and local applicable laws and regulations.

### Patients

Children aged six to 59 months (inclusive), weighing 5 kg or more with fever ≥37.5°C (axillary), who presented to the Ombeyi Dispensary with probable clinical malaria, mono-infection with *P. falciparum* at an asexual parasite density of 1,000-200,000 parasites/μL, and who were able to take drug orally, were eligible for enrolment in the study. Patients were excluded if they had severe and/or complicated malaria [21], including severe anaemia (haemoglobin (Hb) ≤5 g/dL), experienced two or more seizures in the previous 24 hours and hyperparasitaemia (parasites >200,000/μL), or had a general clinical condition requiring hospitalization. Patients who had concomitant infections/disease at the time of presentation, with past or present history of chronic illnesses or any other underlying illness that would compromise the diagnosis and evaluation of the response to the study drug, with a history of allergy to artemisinin, lumefantrine or piperaquine were excluded. Patients undergoing full treatment with other anti-malarial drugs within the previous 14 days were also excluded. Eligible patients were enrolled after the parent/guardian signed the written informed consent.

### Study design

The study comprised a three-day treatment period (day 1 taken as the first day of treatment) and 42-day follow-up. Eligible patients were randomized to one of the two treatment arms (1:1) to receive either AL dispersible (six doses/three days) or DP paediatric (three doses/three days). Patients in the AL dispersible arm with body weight 5–14 kg received one tablet (artemether 20 mg, lumefantrine 120 mg) per dose, and 15–24 kg received two tablets per dose. Patients in the DP paediatric arm received the standard dosage of 2.25 mg/kg and 18 mg/kg per dose of dihydroartemisinin and piperaquine, respectively, rounded up to the nearest half tablet. The drugs were dispersed in a small volume of water or milk and administered by the parents/caregivers under the observation of the study personnel. If there was vomiting within 30 minutes of the dose then the child was re-dosed and if the child vomited the replacement dose, he/she was dropped from the study. All the drug administration occurred in an inpatient setting. Children who developed severe malaria were treated with quinine as per the national guidelines.

Children were admitted to hospital for the first three days for observed treatment and close monitoring. They were evaluated daily in the ward and eight-hourly blood slides were performed until two consecutive negative blood smears for asexual falciparum malaria were obtained. Children were discharged after they had a negative slide and were clinically stable. At discharge, the parent/caregiver completed a questionnaire on the treatment acceptability, and the children were followed up on days 7, 14, 28, 42 and any other day the child was unwell. During the visits, a blood slide was analysed for parasite quantification; blood was collected for a complete blood count (CBC) and a filter paper sample for genotyping in case of parasite re-appearance; physical examination was performed and vital signs, axillary temperature were recorded. Adverse events (AEs) and serious adverse events (SAEs) were recorded and monitored throughout the study.

### Assessments

The primary efficacy assessment included patients with PCR-corrected parasitaemia by day 28, i e, patients with an adequate clinical and parasitological response (ACPR). According to the WHO definition [22], ACPR is the absence of parasitaemia on day 28 or day 42 irrespective of axillary temperature, without previously meeting any of the early treatment failure (ETF), late treatment failure (LTF) or late parasitological failure (LPF) criteria. As per the WHO definition [22], ETF is development of danger signs of malaria or severe malaria on post treatment days 0, 1, 2 or 3, with evidence that the patient is symptomatic and parasitaemic to a greater degree than the value recorded at entry to the trial on day 2; parasitaemic on day 3 greater than 25% of the value recorded on entry to the trial and with axillary temperature ≥37.5°C. LTF is development of danger signs or severe malaria between post-treatment days 4 to 14 inclusive when: a) the patient has not previously met the criteria for ETF and, b) PCR analysis of markers including merozoite surface protein (MSP)-1, MSP-2 and glutamate-rich protein (GLURP) suggests that the parasites are unlikely to be a new infection, with evidence that the patient is symptomatic and any level of *P. falciparum* parasitaemia on any day between 4, 28 or day 42, inclusive with axillary temperature ≥37.5°C in patients not meeting ETF criteria earlier. LPF is presence of parasitaemia on any day between day 7 and day 28 (or day 42) with axillary temperature <37.5°C in patients who did not previously meet any of the criteria of ETF or late clinical failure.

The secondary efficacy and safety assessments included parasitological failure at days 3, 14 and 42, (defined as presence or absence of parasitaemia on assessment), determination of the level of adherence, ease of use, acceptability and adverse events (serious and non-serious) reported as an indicator of safety for both drugs among patients enrolled in the study.

Safety assessments included recording of adverse events (AEs, by system organ class and preferred terms), serious adverse events (SAEs), treatment emergent serious and non-serious adverse events (AEs occurring between day 1 and 35 of treatment period or AEs with unknown onset dates), and collection of clinical laboratory data for haematology and blood chemistry. According to the ICH guidelines [20], AEs are defined as any untoward medical occurrences in a patient administered a pharmaceutical product and which does not necessarily have a causal relationship to the treatment. SAEs are untoward medical occurances that at any dose, result in death, are life threatening, require hospitalization, prolongation of existing hospitalization or result in persistant, and significant disability, or is a congenital anomaly/birth defect. During the study period the area was under indoor residual spraying (IRS) for malaria as a pilot district in the deployment of IRS in high transmission areas by the Division of Malaria Control of the Ministry of Public Health and Sanitation, Kenya.

### Statistical analysis

In this non-inferiority trial, it was estimated that 193 patients per arm were needed to complete the study, assuming that at day 28, the efficacies of AL dispersible and DP paediatric were 90%, with a power of 80%, and a two-sided significance level of 5%. Allowing for a 10% dropout rate, it was calculated that 233 patients per arm (426 in total) had to be randomized. Baseline data were summarized by continuous variables using mean (standard deviation (SD)) if normally distributed, tested using t-test or ANOVA where appropriate or using median and inter-quartile range and non-parametric testing if not normally distributed.

The efficacy variables were analysed on the intent-to-treat (ITT) and per protocol (PP) population. The ITT population included all patients who received at least one dose of study drug. Patients with a major protocol deviation were included in the analyses. The PP population excluded patients with predefined protocol violations and included patients who completed all visits as specified in the protocol; had no major protocol violation with regards to inclusion/exclusion criteria; did not take any prohibited concomitant medications during the treatment period. Patients who were withdrawn from the study due to an AE or lack of efficacy, or who were considered a treatment failure, were included in the PP population.

For the primary efficacy variable, the treatment effect was the difference in efficacy between the two arms at day 28. Missing data was analysed by using complete case analysis wherein patients with missing efficacy data were excluded from the analysis. For secondary efficacy analyses, the treatment effect was the difference in efficacy between the two treatment arms at days 3, 14 and 42. In the analyses of safety data, the biological parameter analysis by treatment regimen was assessed by the mean (95% confidence interval (CI)) change from baseline on day 28. The mean (SD) parameter value at day 28 was presented for each arm. Formal testing for a difference between treatments was done using ANOVA comparing average day 28 values, adjusting for baseline values. Adverse events (both serious and non-serious) have been tabulated using preferred terms by treatment arm in Medical MeDRA V. 12. Failure rate has also been presented based on parasite density at baseline.

## Results

This study was conducted at Ombeyi dispensary in Nyando District, Kenya. The first patient was enrolled on 5 March, 2010 and the last patient completed the study on 30 November, 2011.

### Patient disposition, demographics, and baseline characteristics

**Figure 1:**
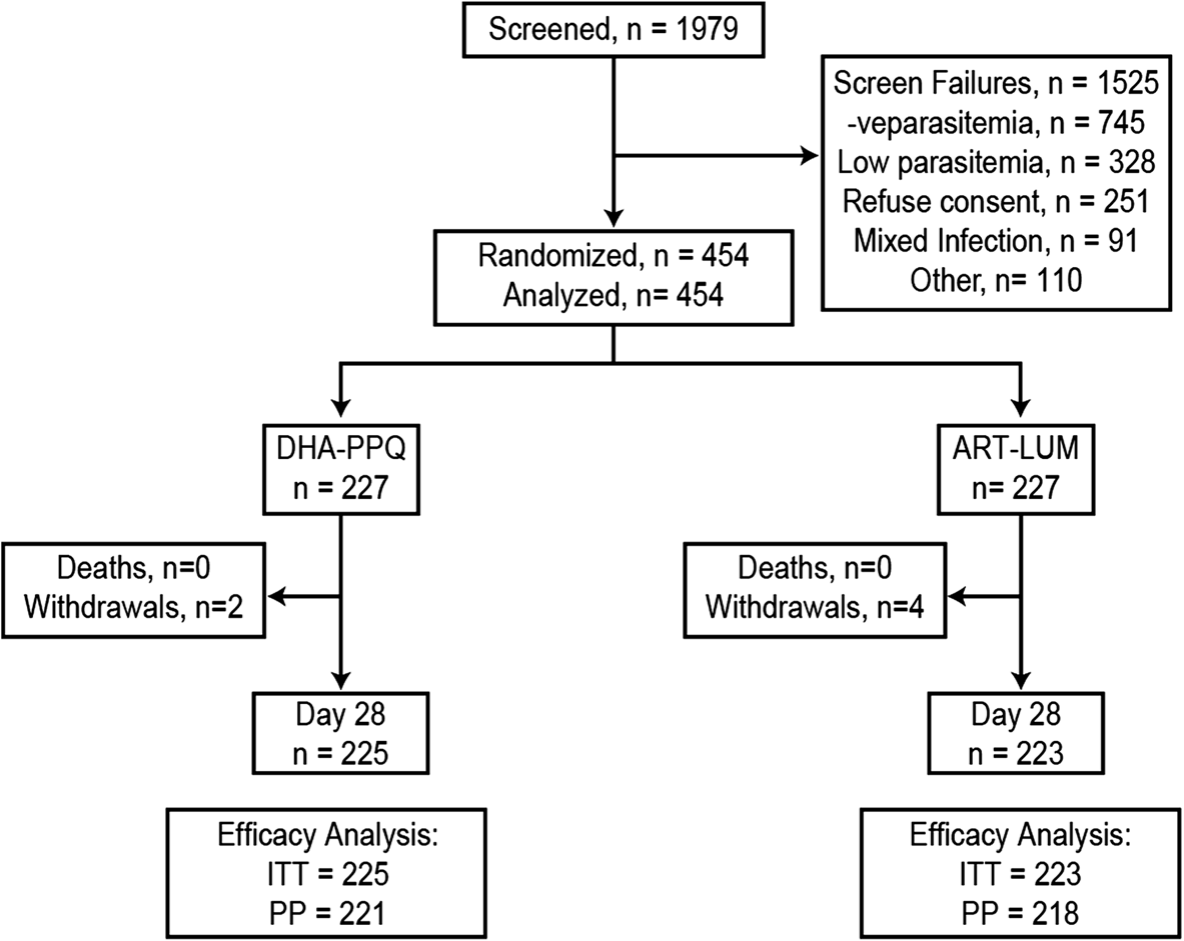
Patient flow.

A total of 1979 children were screened of which 1525 were screening failures, with most being non-malaria cases, low parasite density, mixed species malaria infections and consent refusal (Figure 1). In total, 454 patients were randomized (1:1) to the AL dispersible or DP paediatric treatment arms and 448 (98.6%) completed the study. Two patients receiving DP and four patients receiving AL dispersible treatment were withdrawn from the study by the investigator after day 1 due to inability to tolerate the medication (vomiting; Figure 1). The baseline demographics and clinical characteristics were similar between the two arms (Table 1).

**Table 1:** Patient demographics and baseline characteristics

**Characteristics**	**AL dispersible (n = 227)**	**DP paediatric (n = 227)**	p value*
Age (months)	29.6 (15.2)	32.0 (14.9)	0.094
Weight (kg)	12.2 (3.2)	12.3 (3.2)	0.709
Temperature (°C)	38.3 (1.0)	38.3 (2.1)	0.949
Parasite density (/μL), mean (95% CI)^†^	38,202.2 (32,180.0 to 45,351.3)	38,545.7 (32,615.8 to 45,553.8)	0.941^‡^
Haemoglobin (g/dL)	9.2 (1.9)	9.4 (2.0)	0.293
White blood cells (x10^3^/μL)	9.9 (4.5)	9.5 (3.8)	0.258
Platelets (x10^3^/μL)	134.8 (66.1)	134.9 (67.1)	0.988
Red blood cells (x10^6^/μL)	3.7 (0.9)	3.7 (0.8)	0.764
Neutrophils (%)	41.0 (17.5)	43.1 (17.8)	0.203
Lymphocytes (%)	50.6 (17.2)	48.4 (17.5)	0.182
Monocytes (%)	7.4 (3.0)	7.6 (4.9)	0.559

Data are mean (±SD) unless otherwise specified. AL: Artemether-lumefantrine; DP: dihydroartemisinin-piperaquine; *p value from t-test or Mann–Whitney test; ^†^Geometric mean with its 95% CI; ^‡^p value from t-test on the log parasite density.

**Figure 2:**
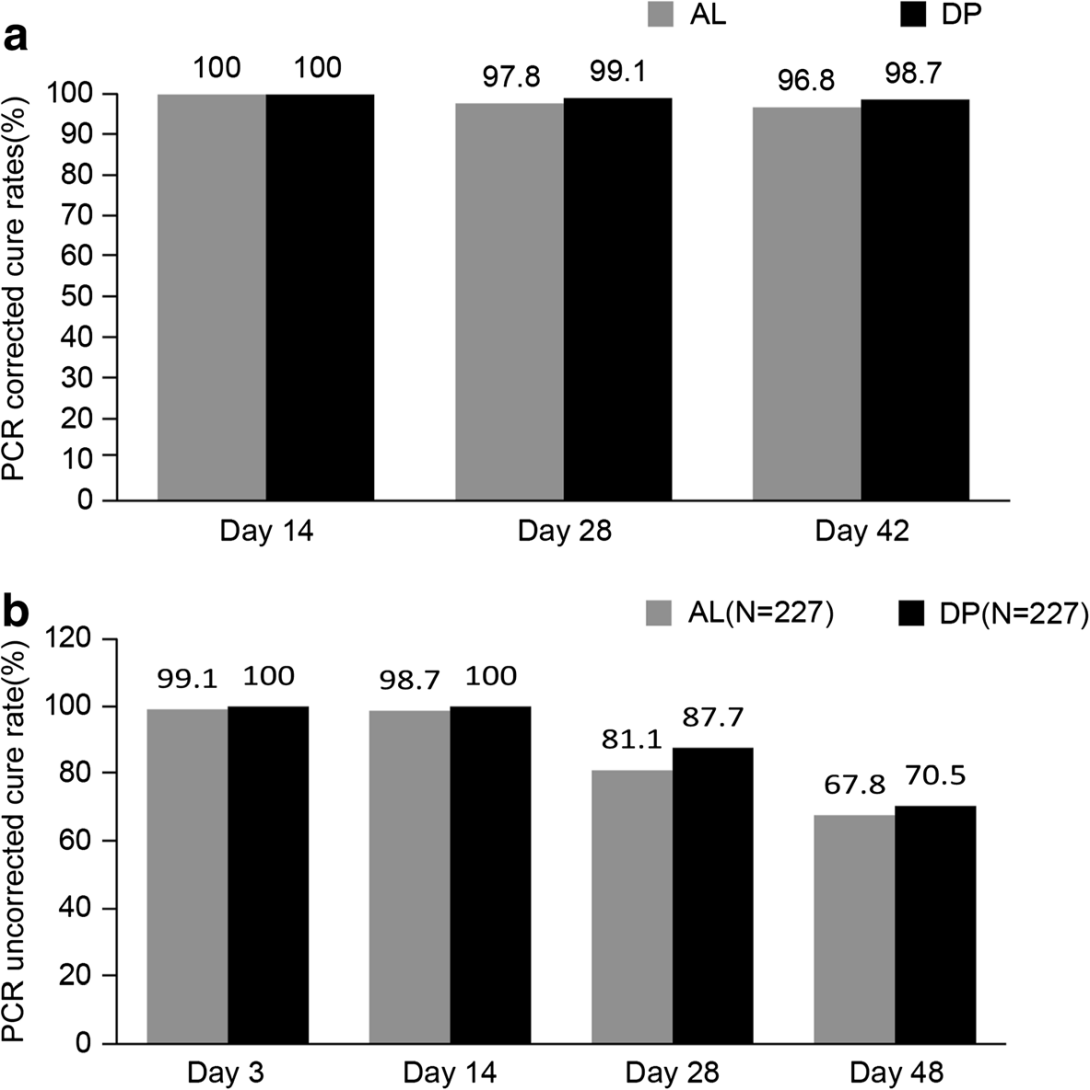
**PCR-corrected and uncorrected cure rates in ITT population. a)** PCR-corrected cure rates in ITT population. **b)** PCR-uncorrected cure rates in ITT population. AL: Artemeter-lumefantrine dispersible; DP: dihydroartemisinin-piperaquine paediatric, ITT: intension to treat.

The corrected ACPR rates in the ITT population were 97.8% (95% CI 94.9-99.3) and 99.1% (95% CI 96.8-99.9) for AL dispersible and DP paediatric, respectively, on day 28 (treatment difference 1.3%, 95% CI: -1.0-3.6%; p = 0.258; Figure 2a). No significant treatment differences were observed for the corrected ACPR rates on days 14 and 42 between the AL dispersible and the DP paediatric arm in the IIT population (Figure 2a). For the uncorrected cure rates, no significant differences were seen on days 3, 14 and 28 and 42 between the AL dispersible and the DP paediatric arm (p > 0.05) for all comparisons (Figure 2b). Similar results were obtained in the PP population for corrected and uncorrected ACPR rates (Figure 2).

**Figure 3:**
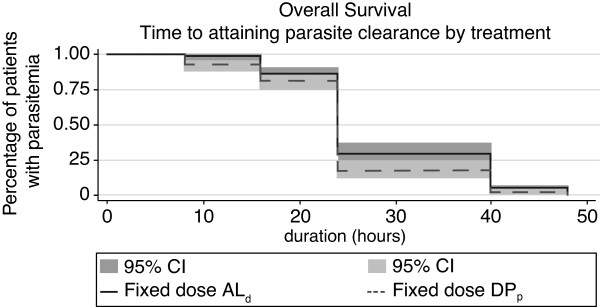
Parasite clearance time following ACT treatment for malaria.

Parasite clearance was rapid, with 90% clearance achieved in 40 hours in both treatment arms (Figure 3), and 50% parasite clearance was achieved within 24 hours in both treatment arms. Treatment failure at day 28 was unaffected by baseline parasite load for both AL dispersible and DP paediatric arms. The treatment failure rate for AL dispersible and DP paediatric was comparable for parasite densities of <50,000/μL and >50,000/μL (23 *vs* 20 for AL dispersible, p = 0.634; 12 *vs* 14 for DP paediatric; p = 0.959; Table 2). Similar results were observed for parasite rates <100,000 and >100,000 (31 *vs* 12 for AL; p = 0.901; 23 *vs* 5 for DP; p = 0.379).

**Table 2:** Effect of parasite load at baseline on failure: day 28

**Parasite load at baseline**	**Failure at day 28**	p-value*	**AL dispersible**	p-value*	**DP dispersible**	p-value*
≤50,000 (n = 223)	37 (16.6%)	0.796	23 (20.5%)	0.735	14 (12.6%)	0.959
>50,000 (n = 219)	34 (15.5%)		20 (18.2%)		14 (12.6%)	
≤100,000 (n = 328)	54 (16.5%)	0.768	31 (19.1%)	0.851	23 (13.9%)	0.484
>100,000 (n = 114)	17 (14.9%)		12 (20.0%)		5 (9.3%)	

*p-value from chi-square/fishers exact test.

### Safety

The overall incidence of AEs was 65.5% (156/238) and 67.5% (156/231) in the AL dispersible and DP arms, respectively (Table 3). The most frequently reported AEs were related to the underlying disease; malaria was reported in both treatment arms (25.6% and 18.2% in the AL dispersible and DP paediatric arms, respectively). Cough was predominant in both treatment arms (15.5% and 17.3% in the AL dispersible and DP paediatric arms, respectively). One patient in the AL dispersible arm had a SAE, severe malaria, not considered by the investigator to be related to the study drug. No deaths were reported during the study. Both treatments were generally well tolerated. The safety profile was comparable for AL dispersible and DP paediatric arms with regard to biological parameters including haemoglobin, white blood cells, platelets, red blood cells (RBCs), lymphocytes and monocytes (Table 4).

**Table 3:** Most frequent adverse events (≥2% in any treatment arm)

**Adverse events**	**AL dispersible (n = 238)**	**DP paediatric (n = 231)**
Patients with at least one adverse event	156 (65.54)	156 (67.53)
Malaria	61 (25.63)	42 (18.18)
Cough	37 (15.55)	40 (17.32)
Anaemia	10 (4.20)	8 (3.46)
Fever	7 (2.94)	14 (6.06)
Tinea capitis	10 (4.20)	12 (5.19)
Rhinitis	4 (1.68)	13 (5.63)
Gastroenteritis	9 (3.78)	5 (2.16)
Loss of appetite	3 (1.26)	6 (2.59)
Otitis media	7 (2.94)	5 (2.16)

AL: Artemether-lumefantrine; DP: dihydroartemisinin-piperaquine.

**Table 4:** Biological parameter changes during treatment

**Parameter**	**AL dispersible**		**DP paediatric**		p-value*
	**N**	**Mean change (95% CI)**	**N**	**Mean change (95% CI)**	
Haemoglobin (g/dl)	178	1.6 (1.3 to 1.9)	185	1.7 (1.4 to 1.9)	0.134
White blood cells (X10^3^/μL)	178	−0.8 (−1.5 to −0.1)	185	−0.5 (−1.0 to 0.3)	0.869
Platelets (X10^3^/μL)	178	88.5 (74.1 to 102.8)	185	88.3 (76.2 to 102.8)	0.926
Red blood cells (X10^6^/μL)	178	0.6 (0.4 to 0.8)	185	0.7 (0.6 to 0.9)	0.394
Neutrophils (%)	178	−14.4 (−17.2 to −11.6)	185	−16.6 (−19.4 to −13.9)	0.857
Lymphocytes (%)	178	14.4 (11.3 to 17.4)	185	18.8 (16.0 to 21.6)	0.081
Eosinophils (%)	178	−0.2 (−0.7 to 0.2)	185	-	-
Basophils (%)	177	−0.02 (−0.10 to 0.07)	185	−0.08 (−0.19 to 0.03)	0.309
Monocytes (%)	177	−0.4 (−0.89 to 0.06)	185	−0.78 (−1.54 to −0.02)	0.633

CI = confidence interval.

A negative value indicates a decrease in day 28 values, on average compared to baseline.

*p-value from ANCOVA test of difference at day 28 between arms adjusting for baseline values.

Tolarability and acceptability assessed by caregiver questionnaire, including general questions with respect to preferred paediatric formulations, are presented in Table 5. Adherence to treatment regimen was higher in the AL dispersible arm (93.6%) compared to DP paediatric (85.6%). Among the 126 patients randomized to receive AL dispersible, 103 (82%) considered it ‘simple’ or ‘very simple’ to use compared with 83 (67%) in the DP paediatric arm. The taste of AL dispersible was ‘liked’ or ‘liked very much’ by 72% of respondents, compared with 56% for DP paediatric. The majority in both groups took the drug with a meal (AL dispersible = 94.4%; DP paediatric = 89.4%), and preferred water to dissolve the tablets (AL dispersible = 94.4%; DP paediatric = 89.4%). In general, caregivers preferred the dispersible tablet formulation (drug given as tablet dissolved in a small volume of water/milk) as compared to a syrup formulation (AL dispersible = 76.8% *vs* 16.8%; DP paediatric = 62.3% *vs* 29.5%).

**Table 5:** Drug questionnaire response

	**AL dispersible N = 126**	**DP paediatric N = 124**	p-value*
Regimen, median number of tablets required	6	4	
Pack available for survey, n (%)	117 (93.6)	107 (85.6)	0.089
**Acceptability and comprehension**			
Health worker explained how to administer drug, n (%)	124 (99.2)	122 (98.4)	0.684
Health worker used pictures to explain how to administer drug, n (%)	123 (99.2)	123 (99.2)	0.321
Pictures helped in understanding how to administer, n (%)	124 (99.2)	124 (99.2)	0.159
Difficulty/Ease of using drug			0.001
Very difficult, n (%)	0	3 (2.4)	
Difficult, n (%)	2 (1.6)	10 (8.1)	
Acceptable, n (%)	19 (15.2)	28 (22.6)	
Simple, n (%)	85 (68.0)	56 (45.2)	
Very simple, n (%)	18 (14.4)	27 (21.8)	
Difficulty/Ease of using drug A compared to B			0.007
Very difficult, n (%)	-	-	
Difficult, n (%)	3 (2.4)	11 (8.9)	
Acceptable, n (%)	25 (20.0)	28 (22.6)	
Simple, n (%)	82 (65.6)	59 (47.6)	
Very simple, n (%)	14 (11.2)	26 (21.0)	
Taste of medicine for child			0.001
Not at all, n (%)	0	4 (3.2)	
Not very much, n (%)	11 (8.8)	24 (19.4)	
Acceptable, n (%)	23 (18.4)	25 (20.2)	
Liked it, n (%)	80 (64.0)	50 (40.3)	
Liked it very much, n (%)	10 (8.0)	20 (16.1)	
Liquid for dissolving tablet			0.301
Water, n (%)	119 (95.4)	115 (92.7)	
Other, n (%)	6 (4.6)	10 (7.3)	
Drug taken with a meal, n (%)	118 (94.4)	110 (89.4)	
Prefer drug given as			0.025
Syrup, n (%)	21 (16.8)	36 (29.5)	
Whole tablet which dissolves in small volume of water/milk, n (%)	96 (76.8)	76 (62.3)	
Injection, n (%)	2 (1.6)	7 (5.7)	
Other, n (%)	6 (4.8)	8 (6.4)	
Prefer other anti-malarias, n (%)	4 (3.2)	5 (4.0)	
**Perceived adverse drug reactions**			
Anything unusual after medication given, n (%)	4 (3.2)	1 (0.8)	
Concomitant medication given, n (%)	3 (2.6)	2 (1.9)	
Adverse reaction solicited, n (%)	2 (1.7)	1 (0.9)	
Caretaker reported ADR to health worker, n (%)	2 (1.7)	1 (0.9)	
ADR/SAE completed based on events seriousness, n (%)	1 (0.9)	1 (0.9)	

AL: artemether-lumefantrine; DP: dihydroartemisinin-piperaquine.

## Discussion

In the present study, AL dispersible and DP paediatric were found to be highly efficacious for the treatment of uncomplicated falciparum malaria in Kenyan children less than five years of age. The 28-day corrected ACPR rates in patients receiving AL dispersible were not significantly different to those seen in patients receiving DP paediatric. The corrected ACPR rates were similar to those previously reported in Kenya and other parts of Africa [19,22]. This shows that despite scaled-up use of AL dispersible in Kenya, the AL is still efficacious as first-line treatment. In contrast to earlier findings [19,23,24], there were no significant treatment differences noted in uncorrected ACPR rates between the AL dispersible and DP paediatric arms at any of the time points assessed, despite the difference in half-life between lumefantrine (four to six days) [25,26] and piperaquine (two to three weeks) [27]. This may be due to the intensity of transmission in this region.

The current study was conducted in children aged between six and 59 months in the meso-endemic area of Kenya, where the malaria prevalence rate has increased from 4% in 2007 to 8% in 2010 [28]. This high-risk population has less likelihood of semi-immunity, and hence the efficacy detected was not greatly influenced by previous exposure to malaria [29].

With widespread adoption and scaled-up use of AL dispersible and other ACT in many sub-Saharan African countries, there is a risk of development of resistance; however, so far parasite resistance has not been reported in this region. In the present study, the majority of the children had parasite clearance within 48 hours with AL and DP and the parasite clearance rate reported here was similar to previously reported rates [30,31]. This demonstrates that the *P. falciparum* parasites in this region are still sensitive to artemisinin derivatives, unlike in some regions of Southeast Asia where delayed response to ACT has been reported [32,33]. This study builds on evidence for clinical practice in Kenya and sub-Saharan African countries that AL dispersible remains efficacious as a first-line treatment for uncomplicated *P. falciparum* malaria.

Overall, both drugs were well tolerated by children. There was a comparable occurrence of AEs in the AL dispersible and DP pediatric arms, with the most commonly reported AEs being malaria and cough, in line with the previously published data [23,34-38]. No deaths were reported. One SAE was reported in the AL dispersible arm (severe malaria) and this was not considered to be related to the study drug. No discontinuations due to the study drug were observed. One limitation of the study was that it was not blinded and there was therefore potential for bias regarding tolerability and efficacy assessments. This was mitigated with a blinded randomization procedure and with the parents being blinded regarding the assigned drug.

Both of the ACT studied here are fixed-dose formulations. AL dispersible is administered twice daily for three days, whereas DP paediatric is administered once daily for three days. AL dispersible should be administered with food or milk [16] to enhance lumefantrine absorption. Although the above facts might suggest that better treatment acceptability and tolerability may be observed with DP paediatric compared with AL dispersible, according to the survey conducted in this study, acceptability and tolerability to treatment regimen was in fact higher in the AL dispersible arm. More respondents considered AL dispersible simple to use and to have a better taste. Most ACT need to be crushed and mixed with water or food. Since they have a bitter taste, children can expectorate the medicine and this may result in them not receiving the full therapeutic dose. With its palatable flavour (‘liked’ or ‘liked very much’ by 72% of respondents), AL dispersible may enhance adherence and improve therapeutic outcomes in children [39,40]. Moreover, it has been demonstrated that with the content of standard African diets or milk optimal efficacy is achieved with fixed-dose AL (Coartem) [16]. Oral bioavalability of AL was shown to increase by 108% when a normal meal was taken close to AL dosing compared to a fasting condition. Another finding of note in the acceptability and tolerability survey was the strong preference expressed for whole tablets, which dissolve in a small volume of water/milk over syrup formulations. Previous research has shown that dry powder formulations intended for suspension in water are more often substandard, relative to tablets [41], and may contain ineffective or incorrect amounts of preservatives [42]. DP may be a good alternative to AL or may have use as rescue medication, and advice should be given regarding the policy and clinical practice for deployment of DP as second-line therapy in the treatment of uncomplicated falciparum malaria in Kenya.

## Conclusions

AL is efficacious and remains the first-line treatment option for uncomplicated falciparum malaria. Both drugs were well tolerated by children. DP has proved to be as efficacious as AL. Parasites in this area remain sensitive to artemisinins.

## Competing interests

None of the authors has affiliation to the manufacturers, and they have declared that they have no competing interests. The drugs were donated by the respective manufacturers.

## Authors’ contributions

BRO designed the research (development of overall research plan and study oversight), conducted the research (data collection and patient care), and analysed the data. KO collected data, was involved in patient care and data analysis. NK collected data and was involved in patient care. JO, GO and CO designed the research plan, collected, and analysed the data. LO, DO and WA designed the research. FE and JJ carried out the molecular studies. RO and EJ designed the study and analysed the data. All authors participated in the preparation of the manuscript. All authors read and approved the final manuscript.
